# A Nurse-Led Self-Management Support Intervention (ZENN) for Kidney Transplant Recipients Using Intervention Mapping: Protocol for a Mixed-Methods Feasibility Study

**DOI:** 10.2196/11856

**Published:** 2019-03-01

**Authors:** Denise Beck, Janet Been-Dahmen, Mariëlle Peeters, Jan Willem Grijpma, Heleen van der Stege, Mirjam Tielen, Marleen van Buren, Willem Weimar, Erwin Ista, Emma Massey, AnneLoes van Staa

**Affiliations:** 1 Section of Nephrology & Transplantation Department of Internal Medicine Erasmus MC, University Medical Center Rotterdam Rotterdam Netherlands; 2 Research Center Innovations in Care Rotterdam University of Applied Sciences Rotterdam Netherlands; 3 Section of Nursing Science Department of Internal Medicine Erasmus MC, University Medical Center Rotterdam Rotterdam Netherlands; 4 Erasmus School Health Policy & Management Erasmus University Rotterdam Rotterdam Netherlands

**Keywords:** chronic kidney disease, evidence-based nursing, self-management, transplantation

## Abstract

**Background:**

Optimal self-management in kidney transplant recipients is essential for patient and graft survival, reducing comorbidity and health care costs while improving the quality of life. However, there are few effective interventions aimed at providing self-management support after kidney transplantation.

**Objective:**

This study aims to systematically develop a nurse-led, self-management (support) intervention for kidney transplant recipients.

**Methods:**

The Intervention Mapping protocol was used to develop an intervention that incorporates kidney transplant recipients’ and nurses’ needs, and theories as well as evidence-based methods. The needs of recipients and nurses were assessed by reviewing the literature, conducting focus groups, individual interviews, and observations (step 1). Based on the needs assessment, Self-Regulation Theory, and the “5A’s” model, change objectives were formulated (step 2). Evidence-based methods to achieve these objectives were selected and subsequently translated into practical implementation strategies (step 3). Then, program materials and protocols were developed accordingly (step 4). The implementation to test the feasibility and acceptability was scheduled for 2015-2017 (step 5). The last step of Intervention Mapping, evaluation of the intervention, falls outside the scope of this paper (step 6).

**Results:**

The intervention was developed to optimize self-management (support) after kidney transplantation and targeted both kidney transplant recipients and nurse practitioners who delivered the intervention. The intervention was clustered into four 15-minute sessions that were combined with regular appointments at the outpatient clinic. Nurses received a training syllabus and were trained in communication techniques based on the principles of Solution-Focused Brief Therapy and Motivational Interviewing; this entailed guiding the patients to generate their own goals and solutions and focus on strengths and successes. Kidney transplant recipients were encouraged to assess self-management challenges using the Self-Management Web and subsequently develop specific goals, action plans, and pursuit skills to solve these challenges.

**Conclusions:**

The Intervention Mapping protocol provided a rigorous framework to systematically develop a self-management intervention in which nurses and kidney transplant recipients’ needs, evidence-based methods, and theories were integrated.

**International Registered Report Identifier (IRRID):**

DERR1-10.2196/11856

## Introduction

Kidney transplantation is the best option for end-stage renal decease. However, kidney transplant recipients need to adhere to a lifelong medication regimen, and optimal self-management is essential for patient and graft survival, reducing comorbidity and health care costs while improving the quality of life [1-6]; this has led to an increasing interest in optimizing patients’ self-management skills [7].

Self-management can be defined as the ability of an individual, in conjunction with family, community, and health care professionals, to manage symptoms, treatments, lifestyle changes, psychosocial, cultural, and spiritual consequences of health conditions to maintain a satisfactory quality of life [8]. Despite the importance of optimal self-management after transplantation, nonadherence to immunosuppressive medication, diet, and exercise have been reported to be relatively high (20%-35%) [9-11]. In addition, recipients report self-management tasks to be challenging, such as adhering to immunosuppressive medication, monitoring symptoms and managing side effects, lifestyle changes, and coping with psychological consequences [12], and report the need for improved self-management support from health care professionals [13-16]. Studies have revealed that self-management support can lead to higher patient well-being and quality of life, improved health, and a decrease in care consumption [3,17,18].

Interventions aimed at optimizing kidney transplant recipients’ self-management are, however, scarce. Furthermore, the existing interventions have a number of limitations [19,20], such as a focus on medication adherence without sufficiently integrating psychosocial and behavioral challenges; insufficient tailoring to individual needs; and lack of theoretical framework and use of evidence-based behavioral change techniques. There is, therefore, a need for the development and testing of better-quality interventions, which improve upon these shortcomings.

An important consideration when developing an intervention is the choice of the health care professional providing self-management support. Traditionally, professionals had a paternalistic approach typified by a directive style rather than shared decision making and a focus on medical issues to the detriment of psychosocial issues [21]; this approach may be less effective in establishing a relationship of trust and promoting behavioral changes [21,22]. Nurse practitioners (NPs) are often key actors in psychosocial support and are in an excellent position to create an environment in which patients feel confident in talking about their concerns [23,24]. A self-management support intervention delivered by NPs may therefore help increase effectiveness. However, little is known about current self-management support practices, attitudes toward self-management support among nurses, and their needs to help improve the support offered.

This study aims to develop a nurse-led self-management support intervention in which the needs of kidney transplant recipients and NPs as well as theory and evidence-based methods, are taken into account; to ensure that these components were incorporated, the Intervention Mapping (IM) protocol was used [25]. The final intervention was called ZENN, an acronym derived from the Dutch translation of self-management after kidney transplantation (ZElfmanagement Na Niertransplantatie).

## Methods

### Intervention Mapping

**Figure 1:**
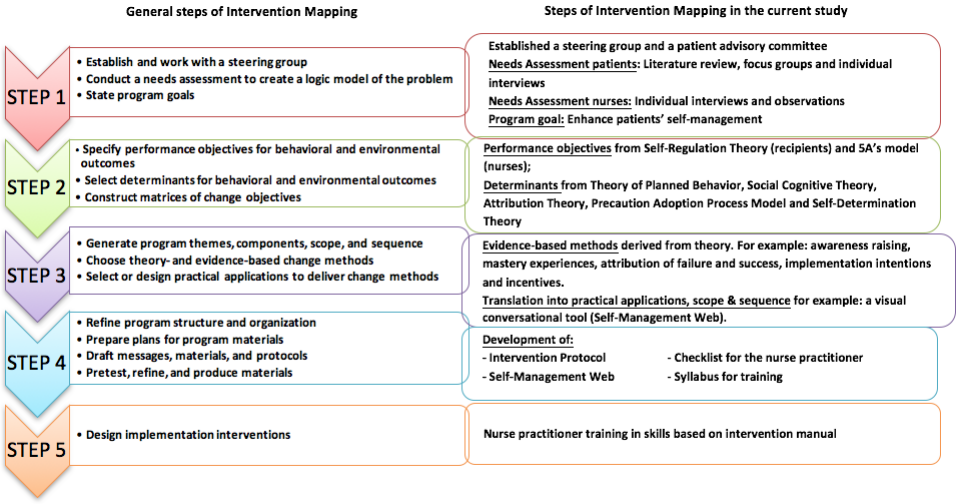
First 5 steps of Intervention Mapping in the ZENN study.

The IM protocol [26] distinguishes 6 steps with corresponding tasks. Here we present the first 5 steps of the IM protocol (Figure 1). In total, the development and implementation of the intervention took 2 years (2015-2017).

### Step 1: Needs Assessment

The first step is the needs assessment—a comprehensive exploration of the health problem and the needs of the targeted population. To ensure that important issues for both kidney transplant recipients and NPs were addressed throughout the process, we established a steering group consisting of NPs, nephrologists, nurse scientists (experts in self-management) and psychologists, alongside a patient advisory committee.

The needs of kidney transplant recipients and NPs regarding self-management (support) were explored in several studies, including a literature review of qualitative studies, interviews, and observations.

#### Assessment of Patients’ Needs

First, we reviewed the qualitative literature on patients’ needs and preferences for self-management support [27]; this review revealed that for patients with chronic conditions, it is important that self-management support is tailored to their individual needs. Furthermore, they need not only information but also instrumental, psychosocial, and relational support. Patients often reported that these needs were unmet because professionals focus on informational and instrumental support alone [27].

Developing a collaborative partnership with shared decision making is key to improving self-management support [27]. This encouraged us to further assess the specific needs, preferences, and challenges with regard to self-management support of kidney transplant recipients through focus groups and individual interviews (n=32) [28]. Participants were recipients from a single transplant center in a university medical hospital in the Netherlands. Results indicated a need for a holistic approach after kidney transplantation. Although recipients were satisfied with the medical care received, psychosocial support focusing on the emotional challenges of living with a transplant was often lacking. Recipients wanted to participate in shared decision making and be collaborators in the process; to achieve this, a relationship of trust was considered essential. This type of support was particularly important in the first year after transplantation. However, one size does not fit all, and self-management support should be adapted to individual needs and circumstances; this was confirmed in a Q-methodological study in the same transplant center, which found differing attitudes toward self-management support [29]. Q-methodology is a qualitative-quantitative method that provides a foundation for the systematic study of peoples’ attitudes toward a certain topic using statements, which are ranked by participants in a quasi-normal grid ranging from completely agree to completely disagree.

#### Assessment of Nurses’ Needs

To explore nurses’ perceptions, attitudes, and potential needs, interviews were held and observations were performed. All participants worked at the same university medical hospital in various outpatient departments. Individual semistructured interviews with nurses and NPs were held (n=27) to investigate nurses’ views on the concept of self-management in general and how these views relate to the self-management interventions they use in clinical practice [30]. Results showed 3 distinct views on self-management support as follows: adhering to a medical regimen; monitoring symptoms; and integrating illness into daily life; only the last viewpoint reflected a holistic approach with the nurse focusing on coaching. Medical management was the focus of self-management for many nurses. The lack of attention for psychosocial aspects may be attributed to a lack of confidence, skills needed to address psychosocial issues, or available tools or interventions that limit them in offering psychosocial support. Providing training or practical interventions protocols or tools for holistic self-management support could partially resolve this problem by giving nurses resources to encourage self-management effectively.

To more objectively assess NPs’ roles and skills in outpatient consultations and how this compares with their perception of their responsibilities for patients with chronic conditions, NPs (n=5) were observed during daily practice [31]. Although NPs reported that they considered building a relationship with their patients of utmost importance, their consultations were mostly based on a conventional medical model of medical history taking. Little attention was paid to the social, psychological, and behavioral dimensions of illness. Finally, a realist review of the literature was conducted to understand how nurse-led interventions that support self-management of patients with chronic conditions work and in what context they work successfully. Interventions focusing on intrinsic processes were found to be the most effective, as opposed to focusing solely on education [32]. Textbox 1 outlines the main findings from the needs assessment.

**Summary of findings from the needs assessment [ 27- 32].**

- Patients’ needs assessment
  - Medical and psychosocial issues should both be addressed; attention to psychosocial needs often lacking
  - Tailoring of support to specific needs and preferences is important to patients
  - Self-management support most needed first-year posttransplant
  - Shared decision making is preferred

- Nurses’ needs assessment
  - Nurses place emphasis on medical management to the detriment of psychosocial management
  - Nurses focus on education rather than on patient empowerment and coaching
  - Nursing interventions focusing on intrinsic processes are more successful in promoting self-management

#### Program Goals

Based on the needs assessment described above, we developed a nurse-led self-management support intervention that included the following key elements: a general, open structure that leaves room for individual preferences and tailoring of support; a holistic approach encompassing medical, emotional, and social self-management challenges; promoting shared decision making between nurses and patients; and patient empowerment by supporting self-efficacy and intrinsic motivation. The overall goal of the intervention is for kidney transplant recipients to enhance their self-management skills to integrate their treatment and life goals and subsequently optimize their quality of life and health-related outcomes. In addition, we aimed to improve NPs’ skills to optimize self-management support.

### Step 2: Matrices of Change Objectives—Kidney Transplant Recipients

**Figure 2:**
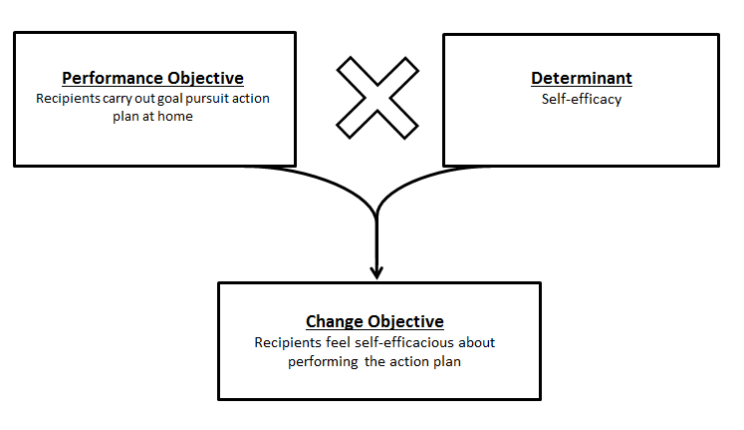
Creation of Change Objectives.

The second step of IM links the overall goals of the intervention to concrete actions by stating change objectives (COs); COs specify who and what will change because of the intervention. To generate COs, we combined performance objectives (POs) and the relevant determinants into a matrix. Figure 2 shows an example of combining a PO with a determinant to obtain a CO. DB and JWG formulated the COs and discussed these with EM for fine-tuning. After this process of revision, 74 COs were formulated and integrated into the intervention.

#### Performance Objectives

The overall program goal was translated into performance objectives (POs) that specify the behavioral actions the target groups need to perform to change behavior successfully. The target groups were kidney transplant recipients and NPs. Optimizing self-management after kidney transplantation requires intrinsic processes (eg, motivation and self-efficacy [33]) and long-term skills to establish and maintain behavior change and also abilities to adapt behavior when circumstances change. Well-developed self-regulation skills are supportive in performing these tasks. Therefore, the specific behavioral actions that contribute to the overall goal of the intervention were specified in POs based on the principles of self-regulation theories [34]. Studies on other chronic illnesses showed that interventions based on self-regulation theories could improve behavioral outcomes [35-37]. Overall, 8 POs were defined, including goal setting, planning, self-monitoring, feedback, and relapse prevention (Textbox 2).

#### Determinants

After the definition of POs, we explored which determinants were associated with the performance of the desired behavior, as stated in POs. The determinants were selected from the following health behavioral change theories: Self-Regulation Theory [34]; Theory of Planned Behavior [38]; Social Cognitive Theory [39]; Attribution Theory [40]; Relapse Prevention Theory [41]; Precaution Adoption Process Model [42]; and Self-Determination Theory [43]. Table 1 shows examples of the COs afor NPs for recipients derived from combining POs and determinants.

### Step 2: Matrices of Change Objectives—Nurse Practitioners

#### Performance Objectives

POs for NPs were also guided by the Self-Regulation Theory. In addition, the intervention focused on 3 components of the Five A’s model of behavior counseling [44] were incorporated, namely assessing behavior, beliefs, and motivation, agreeing with patients on realistic goals, and assisting to anticipate barriers and develop a specific action plan. The other two components of the 5A’s model (advising and arranging) were not an integral part of the intervention because they are less in line with the focus on patient empowerment. To achieve the overall program goal and considering the needs assessment, 2 POs for NPs were formulated (Textbox 2).

#### Determinants

The determinants deemed most pertinent in predicting these POs for NPs were knowledge, skills, social and professional role and identity, self-efficacy, attitude, and outcome expectations. Table 2 shows examples of COs derived from combining POs and determinants. The full COs matrices are available on request.

**Performance objectives for kidney transplant recipients and nurse practitioners.**

- Kidney transplant recipients
  - Recipients decide to improve their self-management on medical or emotional tasks they perceive as challenging
  - Recipients set, at least, one SMART goal
  - Recipients make an action plan to actively pursue and attain their chosen goal, taking into account possible facilitators, barriers, and resources
  - Recipients carry out their goal-pursuit action plan at home
  - Recipients monitor their goal-pursuit behavior in daily life
  - Recipients evaluate their progress with NPs
  - If successful, recipients maintain their new behavior or set a new goal
  - If unsuccessful, recipients adjust their goal, action plan, or outcome expectations
  - Recipients can cope with relapse and reinitiate goal pursuit
  - Recipients can generalize learned self-management skills to new goals

- Nurse Practitioners
  - The nurse practitioners (NPs) carry out the intervention during their consultations with recipients included in the study
  - The NPs assess whether recipients perceive medical, social, or emotional tasks as challenging
  - When recipients indicate that there is a problem in a specific life area, NPs stimulate and guide recipients to set a SMART goal to solve the problem and agrees with the recipient on the goal
  - NPs stimulate and assist recipients to make and implement action plans for attaining their goals
  - NPs encourage recipients to monitor and evaluate their progress toward goal attainment
  - NPs stimulate recipients to maintain goal pursuit or adapt goals or action plans
  - NPs help recipients to anticipate relapse and discuss relapse prevention
  - NPs help recipients to generalize learned techniques to new problems and goals
  - NPs focus on the positive desired outcomes rather than on the negative aspects of living with a kidney transplant

**Table 1:** Examples of change objectives for kidney transplant recipients derived from combining the performance objectives and determinants.

Performance objectives	Behavioral determinants							
	Awareness	Attitude	Self-efficacy		Autonomous motivation	Social support	Commitment	Skills
Recipients decide to improve an aspect of their life	Become aware of and acknowledge improvement is possible in one or more areas in their life; Are aware of the discrepancy between the desired and current situation	Have stronger positive feelings toward improving self-management than negative		Feel able to improve this aspect of their life	Are intrinsically motivated to improve aspect of life	N/A^a^	N/A	N/A
Recipients set, at least, one SMART goal	Are aware of the desired outcome	Have positive feelings toward the goal		Formulate a goal that they feel self-efficacious about	N/A	N/A	N/A	Are capable of setting a SMART goal
Recipients make an action plan to attain and actively pursue their chosen goal	Are aware of possible habits, facilitators, barriers, and resources	Have positive feelings toward the action plan		Draw up an action plan they feel able to carry out	N/A	Consider possible social support when making an action plan	N/A	Are capable of making an action plan in which facilitators, barriers, habits, and resources are considered
Recipients carry out their goal-pursuit action plan at home	N/A	Have stronger positive feelings toward carrying out the plan than negative		Feel able and self-efficacious about performing the action plan	Are intrinsically motivated to carry out an action plan	Use their social resources according to plan	Show commitment to pursuing the behavior in daily life	N/A

^a^N/A: not applicable.

### Step 3: Theory-Based Methods and Practical Strategies

Step 3 aims to identify and select theory-based methods and translate these into practical strategies to influence each determinant to achieve the CO; for example, modeling (method) can be used to influence self-efficacy (determinant) by showing videotaped demonstrations of other patients performing self-management tasks (practical application). Methods and practical applications were reviewed and discussed with the steering group and patient advisory committee. From the methods identified, we selected applications for inclusion in the intervention based on the feasibility and the needs identified in Step 1.

Techniques from Motivational Interviewing [45] were used to promote motivation. The principles of Solution-Focused Brief Therapy (SFBT) [46] were used for the goal and action-oriented COs. SFBT is goal-directed, future-focused, and addresses solutions rather than problems. These key concepts make SFBT particularly useful to actively involve patients during nursing consultations. Furthermore, the social cognitive theories from which determinants of POs were selected were the source of behavioral change methods. The methods were translated into practical applications that were integrated into the intervention protocol. Table 3 shows examples of the theoretical methods and practical applications incorporated into the intervention.

### Step 4: Program Production

In Step 4, the actual program was developed; this step contains the determination of program components, the creation of the program scope and sequence, and the development of program materials. Representatives of the steering group and patient advisory committee were presented the concept program and their feedback guided final adjustments.

#### Intervention Scope

The main theme of the program is optimizing self-management based on the principles of self-regulation theories—evaluating areas of life, establishing and setting goals, planning and preparing strategies for achieving the personal goals and actively pursuing goals, monitoring and evaluating goal progress, and preparing strategies for relapse prevention. Throughout the intervention, these steps are combined with the principles of SFBT to stimulate kidney transplant recipients to generate solutions rather than focusing on their problems.

**Table 2:** Examples of change objectives for nurse practitioners derived from combining the performance objectives and determinants.

Performance objectives	Behavioral determinants						
	Awareness	Knowledge	Skills	Self-efficacy	Attitude		Professional role and identity
NPs^a^ carry out the intervention during their consultations with recipients who have been included in the study	Are aware of benefits using the intervention protocol	Know how to use intervention protocol and when to use which techniques	Have skills (ie, conversational and motivational techniques) to carry out the intervention	Feel self-efficacious to carry out the intervention	Have a stronger positive feeling toward carrying out the intervention than negative	Deem self-management support and carrying out the intervention as part of their professional role	
NPs assess if recipients experience challenges or problems in several areas of life	Become aware of problems in recipients’ life on other than medical domains and the benefits of assessing psychosocial areas	N/A^b^	Have skills to assess and discuss psychosocial and medical aspects	Feel self-efficacious about assessing and discussing psychosocial and medical aspects	Have stronger positive feelings about assessing psychosocial and medical aspects than solely assessing medical aspects	N/A	
When recipients indicate that there is a problem in a specific life area, NPs stimulate recipients to set a SMART goal and agree with recipients on the goal	N/A	Know how to set a SMART goal together with the recipient	N/A	Feel self-efficacious about assisting recipients in setting a SMART goal	N/A	N/A	
NPs assist and stimulate recipients to make and implement action plans for attaining their goals	N/A	Know how to assist the recipient to make an action plan which is achievable	N/A	Feel self-efficacious about assisting recipients in making an action plan	N/A	N/A	

^a^NP: nurse practitioner.

^b^N/A: not applicable.

**Table 3:** Examples of the theoretical methods and practical applications incorporated into the intervention.

Change objectives	Determinants	Theoretical methods	Practical application and strategies
Recipient becomes aware of and acknowledges problems in various areas of life	Awareness (Precaution Adoption Process Model or Theory of Planned Behavior)	Awareness raising providing feedback using visualization	Recipients evaluate their life areas based on the Self-Management Web.
NPs^a^ become aware of problems in recipients’ life on other than medical domains and the benefits of assessing psychosocial areas	Awareness (Precaution Adoption Process Model or Theory of Planned Behavior)	Awareness raising providing feedback using visualization	Self-Management Web: NPs help assess recipients’ life based on the Self-Management Web
Recipients belief in their capabilities to optimize self-management behavior	Self-efficacy (Social Cognitive Theory)	Mastery experiences; Attribution of failure and success	Recipients are asked to evaluate and appoint successes to stable, internal factors, and failure to external, unstable factors. When a recipient experiences success, an NP will emphasize the role of the recipient in the success.
NPs feel self-efficacious about carrying out intervention	Self-efficacy (Social Cognitive Theory)	Modeling	NPs receive training in which they practiced delivery using role-plays
Recipients implement new actions to reach goals and break through habits	Habits (Theory of Automatic Behavior)	Implementation intentions	Recipients need to specify if-then, when, where, how, what and where they are going to perform goal-related actions

^a^NP: nurse practitioner.

#### Intervention Sequence

**Figure 3:**
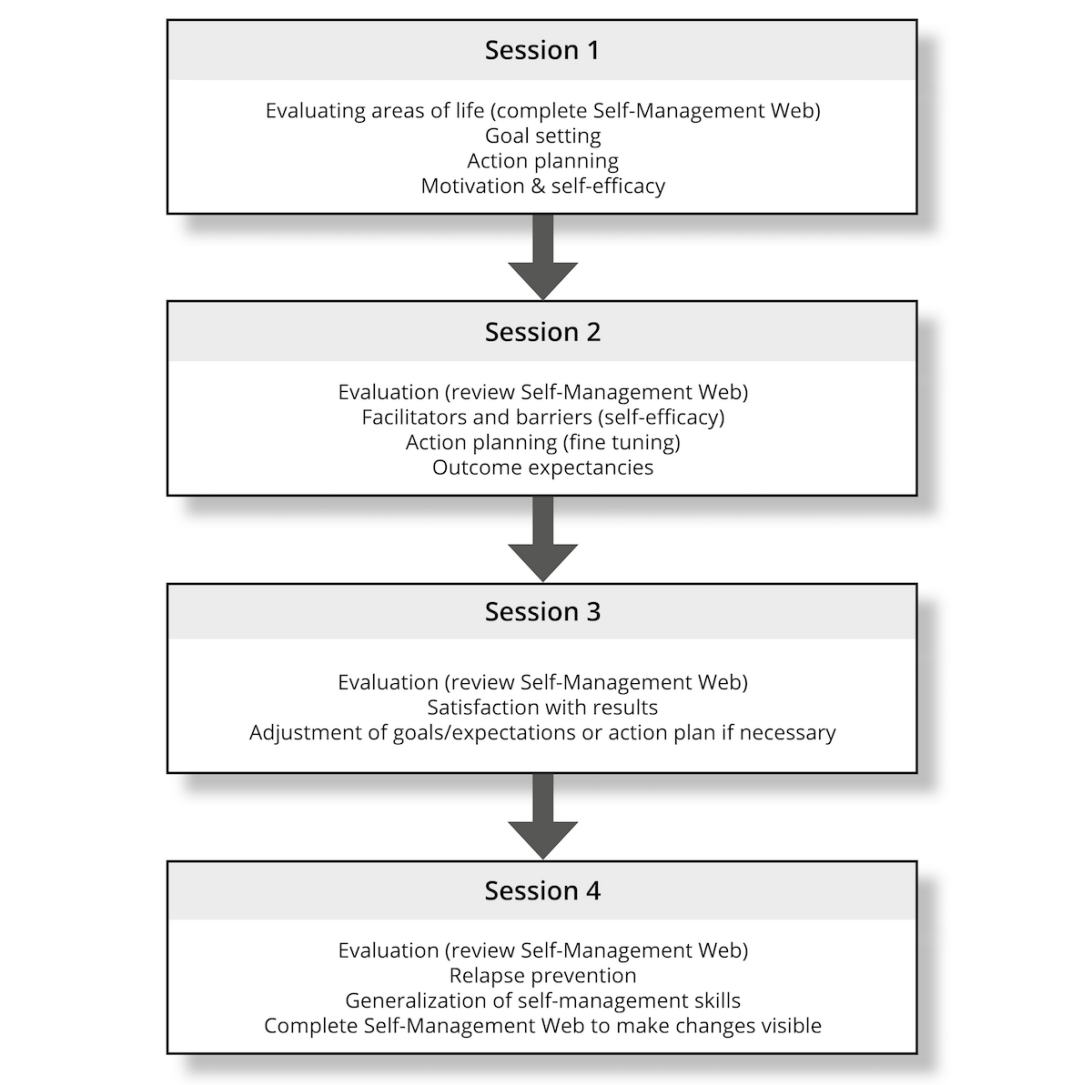
Content of sessions 1-4.

The final program consists of four 15-minute sessions with an NP combined with regular appointments in the outpatient clinic. The frequency of intervention sessions is determined by the frequency of consultations within standard care. Therefore, the period between the sessions can range from 2 weeks to several months. If the period between sessions 1 and 2 is over 1 month, a telephone consultation with the NP is scheduled. During the first session, the emphasis is on assessment—raising awareness, evaluating areas of life, goal setting, and preliminary preparation of an action plan. In addition, motivation and self-efficacy are discussed using visual analogue scales ranging from 0 to 10. The second and third sessions are used to monitor and evaluate the progression on goal attainment during the past weeks and discuss outcome expectations. Throughout the second and third session, the action plan is further customized; self-efficacy is positively encouraged, and outcome expectations are discussed. During the fourth session, goal progress, relapse prevention, and generalization of learned skills to other challenges are discussed (Figure 3).

**Figure 4:**
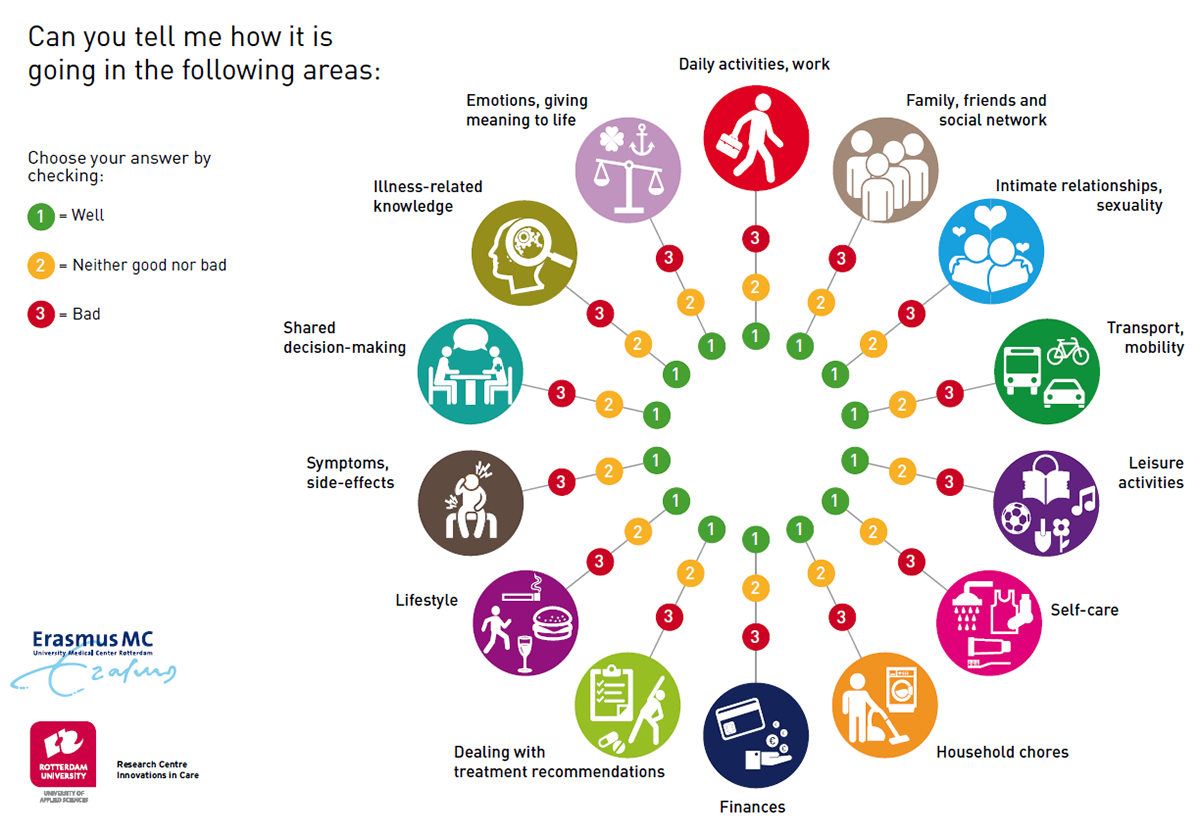
Self-Management Web.

#### Self-Management Web

A visual communication aid called the Self-Management Web (Figure 4) was developed to facilitate the achievement of the first CO (the recipient becomes aware of and acknowledges problems in various areas of life). The Self-Management Web is used to standardize the assessment of 14 life areas and offer a visual overview to guide the conversation between the professional and patient. The Self-Management Web was developed on the basis of the needs of patients with a chronic condition in general and can be used with a variety of patient populations; this tool ensures a holistic view, because multiple areas of life are represented in the Web, and enhances intrinsic motivation because patients determine the area they prefer to focus on. The discussion about goals results in shared decision making between nurses and recipients.

During the first session, NPs encourage kidney transplant recipients to evaluate their life domains and assess if they are doing well (1, green), neither good nor bad (2, orange), or bad (3, red) on each domain. Kidney transplant recipients mark the answer on the Web to visualize domains with difficulties, which contributes to awareness. When kidney transplant recipients report a 2 or 3, an NP asks open questions to clarify the problem. When multiple areas are rated as “bad,” the NP invites the kidney transplant recipient to prioritize and select the area of life he or she wants to work on after which other steps of the intervention are carried out.

#### Intervention Materials

Prior to beginning the intervention, nurses were trained in delivery. A training syllabus was developed, which NPs received before the training. An intervention protocol was written for NPs, containing specific guidelines per session on how to approach kidney transplant recipients and which topics to discuss with suggestions on how to phrase specific questions. To support the implementation and adherence to the protocol, a checklist was developed for NPs to report on the steps executed per session per recipient.

### Step 5: Adoption and Implementation

The effectiveness of an intervention is partially attributable to the quality of the implementation. To promote implementation and ensure fidelity to the intervention, NPs received 2 training sessions before the implementation of the intervention. During the implementation phase, NPs received booster sessions. The training was provided by an experienced psychotherapist (AvtS) and a psychologist (DB).

The training had a dual-purpose; on the one hand, it comprised an explanation on how to carry out the intervention protocol and on the other hand, NPs were trained in using techniques from SFBT and Motivational Interviewing. The training was divided over two 3-hour sessions. After explaining the theories on which the intervention was based and techniques to be used during consultations, trainers performed a role-play to show the steps (modeling). Subsequently, NPs were invited to participate in role-plays with trainers (mastery experiences). Anticipated problems were thoroughly discussed. At the end of the training, the topics discussed were summarized, and the training was evaluated.

Throughout the implementation period, NPs received booster sessions during which problems encountered could be discussed and techniques practiced. Furthermore, video recordings were made as part of the evaluation of the intervention. NPs received feedback based on the video recordings.

### Step 6

In a mixed-methods design, feasibility and preliminary effects of this intervention are being assessed. The outcomes of this step fall outside the scope of this study.

## Results

The enrollment for the feasibility study was completed in March 2018. Data analysis is currently underway and the first results are expected to be published in 2019.

## Discussion

### Principal Findings

The development of the current intervention responds to the need for practical and effective interventions to optimize self-management support after transplantation, in which tailoring, a holistic approach, shared decision making, and patient empowerment is incorporated. In addition, this intervention is in line with the vision of the World Health Organization, which stipulates that the health care system should be addressed when improving self-management support [47] and with recommendations regarding enhancing self-regulation skills among kidney transplant recipients for optimizing the psychological well-being [48].

Although evidence indicates the importance of anticipating the individual needs of each patient to enhance effectiveness, most current interventions fail to do so [19,20,49]. It has been suggested that variance in the effectiveness of self-management support could be attributed to the mismatch between the individuals’ needs and the offered intervention [50]. To improve the fit, the Self-Management Web was used to assess in which areas of posttransplant life recipients were experiencing challenges. The Self-Management Web was developed as part of a consortium on self-management and based on the needs of patients with a chronic condition in general and is therefore applicable to a variety of patient populations. Patients participated in the development and pilot evaluation. The use of the Self-Management Web ensured the standardization of the assessment of multiple areas of life while allowing room for a personalized approach.

In addition, our intervention responded to the tendency for self-management support interventions to focus mainly on medical management to the detriment of psychological and social aspects; this emerged from the needs assessment wherein recipients reported the need for psychosocial support in addition to medical guidance, whereas nurses and NPs acknowledged the shortcomings of their current approach. Studies have shown that psychosocial (eg, depression, anxiety) and behavioral factors could negatively affect self-management and are therefore important targets for self-management support interventions [19,20,33,49].

Furthermore, it has been suggested that interventions should be developed on the basis of theory and evidence-based methods [19,20,25,47]. There is an increasing emphasis on reporting specific behavioral change techniques used in interventions to increase the quality and replicability [51]. The IM protocol helped to integrate theory and evidence-based methods as well as the needs of kidney recipients and nurses into the intervention. Behavioral science offers several useful theories and strategies that enhance the effectiveness of interventions used in health behaviors [47]. Our realist review demonstrated that self-management support interventions focusing on intrinsic processes were most successful in the behavioral change [52]; this emulates earlier authors who have emphasized that education alone is insufficient for health behavioral changes. Examples of these processes were self-efficacy and (intrinsic) motivation, which were in the backbone of the current intervention. The Self-Management Web provides the basis on which important personal goals can be set, which ensures intrinsic motivation. Self-Determination Theory [53] stipulates that intrinsic motivation is an important factor for effective behavioral changes [43]. The intervention protocol encourages motivation during the intervention and also emphasizes increasing self-efficacy. Studies among kidney transplant recipients have stipulated the importance of promoting self-efficacy when supporting self-management in kidney transplant recipients [3,18]. In summary, the strengths of the intervention include tailoring, a holistic approach, focus on intrinsic processes, and promotion of shared decision making.

### Limitations

Although the intervention is based on health behavioral change theories and the methods incorporated are evidence-based, this does not necessarily guarantee the effectiveness in the context of kidney transplantation. Because recipients determine the goals according to their needs and preferences, improving adherence or lifestyle is not always chosen. Goals attained in the intervention maybe too far removed from the health domain to directly relate to positive health outcomes. In contrast, one could also argue that problems in life areas other than health often impact health-related issues and thus self-management owing to the stress they generate. The effectiveness of the intervention is currently under investigation and results will be presented and discussed elsewhere.

## Abbreviations

CO: change objective
IM: intervention mapping
NP: nurse practitioner
PO: performance objective
SFBT: Solution-Focused Brief Therapy
